# Advance in hyperbaric oxygen therapy in spinal cord injury

**DOI:** 10.1016/j.cjtee.2023.05.002

**Published:** 2023-05-12

**Authors:** Antonio Siglioccolo, Renato Gammaldi, Veronica Vicinanza, Alessio Galardo, Vittorio Caterino, Salvatore Palmese, Carmine Ferraiuoli, Alessandro Calicchio, Antonio Romanelli

**Affiliations:** a Department of Anaesthesia and Intensive Care Unit, Azienda Ospedaliero Universitaria “San Giovanni Di Dio e Ruggi D'Aragona”, Via San Leonardo, 84125, Salerno, Campania, Italy; b Department of Diving and Hyperbaric Medicine, Azienda Ospedaliero Universitaria “San Giovanni Di Dio e Ruggi D'Aragona”, Via San Leonardo, 84125, Salerno, Campania, Italy; c Resident in Anaesthesia and Intensive Care, “Federico II” University, Via Sergio Pansini, 80131, Naples, Campania, Italy

**Keywords:** Spinal cord injury, Hyperbaric oxygen therapy, Randomised controlled trial, Protocol

## Abstract

Spinal cord injury (SCI) is a severe lesion comporting various motor, sensory and sphincter dysfunctions, abnormal muscle tone and pathological reflex, resulting in a severe and permanent lifetime disability. The primary injury is the immediate effect of trauma and includes compression, contusion, and shear injury to the spinal cord. A secondary and progressive injury usually follows, beginning within minutes and evolving over several hours after the first ones. Because ischemia is one of the most important mechanisms involved in secondary injury, a treatment to increase the oxygen tension of the injured site, such as hyperbaric oxygen therapy, should theoretically help recovery. Although a meta-analysis concluded that hyperbaric oxygen therapy might be helpful for clinical treatment as a safe, promising and effective choice to limit secondary injury when appropriately started, useful and well-defined protocols/guidelines still need to be created, and its application is influenced by local/national practice. The topic is not a secondary issue because a well-designed randomized controlled trial requires a proper sample size to demonstrate the clinical efficacy of a treatment, and the absence of a common practice guideline represents a limit for results generalization. This narrative review aims to reassemble the evidence on hyperbaric oxygen therapy to treat SCI, focusing on adopted protocols in the studies and underlining the critical issues. Furthermore, we tried to elaborate on a protocol with a flowchart for an evidence-based hyperbaric oxygen therapy treatment. In conclusion, a rationale and shared protocol to standardize as much as possible is needed for the population to be studied, the treatment to be adopted, and the outcomes to be evaluated. Further studies, above all, well-designed randomized controlled trials, are needed to clarify the role of hyperbaric oxygen therapy as a strategic tool to prevent/reduce secondary injury in SCI and evaluate its effectiveness based on an evidence-based treatment protocol. We hope that adopting the proposed protocol can reduce the risk of bias and drive future studies.

## 1 Introduction

Spinal cord injury (SCI) represents a severe lesion comporting a wide range of motor, sensory and sphincter dysfunctions, with abnormal muscle tone and pathological reflex. The most common causes of SCI are vehicle accidents, falls, neoplasia, infection, and other traumatic events.^1^ In the United States, the incidence of SCI is 17,000 people annually, with a prevalence of 282,000 people. The average age is 42 years, and the male gender showed a 4-fold higher incidence than the female.^2^, ^3^, ^4^ Despite advances in understanding the pathogenesis and early recognition and treatment improvements, SCI results in severe and permanent disability, representing a costly problem for society, with high direct medical expenses over the lifetime of a patient.^5^

Most SCIs are associated with injury to the vertebral column, represented by bone fractures, joint dislocations, tearing of a ligament, and disruption and herniation of the intervertebral disc.^5^ From a physiopathological point of view, SCI is mediated by 2 mechanisms, primary and secondary injury. The primary injury is caused by the direct compression, contusion, and shear to the spinal cord and is the immediate effect of trauma. Subsequently, the secondary injury usually follows within minutes and evolves over several hours.^5^^,^^6^ Despite the processes propagating secondary injury being complex and not still wholly understood, possible mechanisms include ischemia, hypoxia, inflammation, oedema, excitotoxicity, ions alteration, and apoptosis.^4^ In patients with an incomplete SCI, the development of secondary injury is sometimes clinically manifest by neurologic deterioration over the first 8 − 12 h after trauma. The spinal cord oedema develops within hours, with a peak between the 3rd and 6th day after the injury, and begins to recede after the 9th day, gradually replaced by central hemorrhagic necrosis.^7^

Because ischemia represents the most crucial mechanism involved in the secondary injury, a treatment to increase the oxygen tension in the injured site can theoretically help recovery.^8^^,^^9^ Hyperbaric oxygen (HBO) therapy is commonly used to treat hypoxic diseases by administering pure or high-concentration oxygen with a mask or in an environment with over 1.4 atmospheric pressure in a hyperbaric chamber. Holbach et al.^10^ were the first to show that HBO therapy could improve postoperative dysfunction in patients with SCI. A meta-analysis concluded that HBO might be useful for clinical treatment as a safe, promising and effective choice.^11^ HBO therapy mainly limits secondary injury when appropriately started, but useful and well-defined protocols/guidelines still need to be created, and its application is influenced by local/national practice. The topic is not a secondary issue because a well-designed randomized controlled trial (RCT) requires a proper sample size to demonstrate the clinical efficacy of a treatment, and the absence of a common practice guideline represents a limit for results generalization.^11^

With this narrative review, we summarize the rationale for using HBO therapy to treat SCI and reassemble the available evidence, focusing on adopted protocols in the studies and underlining the critical issues. Furthermore, we tried to elaborate on a protocol with a flowchart for an evidence-based HBO treatment.

## 2 Potential mechanisms of HBO in SCI and current literature evidence

Ischemia is one of the most important mechanisms involved in secondary injury after SCI. A treatment modality able to increase the oxygen tension of the injured spinal cord should help improve recovery. Most oxygen in arterial blood is carried by haemoglobin, and only a smaller fraction is dissolved in plasma. HBO can increase the amount of dissolved oxygen, creating a larger pressure gradient that can drive oxygen into ischemic tissue. Although an adequate discussion of the mechanisms of HBO in SCI is beyond this paper, experimental data suggest that HBO therapy can exert neuroprotective effects involving multiple mechanisms: decreasing apoptosis, reducing oxidative stress, diminishing inflammation, promoting angiogenesis, reducing spinal cord oedema, and increasing autophagy.^4^

While the application of HBO therapy to treat SCI has a long history, its therapeutic effects in humans are still controversial. Huang et al.^11^ performed a meta-analysis including all RCTs, written in English or Chinese, performed in patients with SCI and treated by HBO therapy. The primary outcomes analysed were American Spinal Injury Association (ASIA) motor and sensory scores, modified barthel index (MBI), Hamilton depression scale (HAMD) and Hamilton anxiety scale (HAMA). The secondary outcomes were the activity of daily living and psychological status. Mean difference (MD) and 95% confidence intervals (*CI*) were computed.

Eleven RCTs were included, 2 English and 9 Chinese. The main results were that HBO therapy significantly improved motor (evaluated in 10 trials, MD = 15.84, 95% *CI:* 9.04 − 22.64, *I*^*2*^ = 87%) and sensitive (evaluated in 6 trials, MD = 66.30, 95% *CI:* 53.44 − 79.16, *I*^*2*^ = 95%) ASIA scores, MBI score (evaluated in 2 trials, MD = 13.80, 95% *CI:* 10.65 − 16.94, *I*^*2*^ = 0%) and reduced HAMD (evaluated in 2 trials, MD = -3.74, 95% *CI:* -5.82 to -1.65, *I*^*2*^ = 90%) and HAMA (evaluated in 2 trials, MD = -2.37, 95% *CI*: -2.72 to -2.02, *I*^*2*^ = 0%).

However, the high risk of bias and heterogeneity limits the generalization of the results. For example, 5 studies performed HBO therapy with an oxygen mask, while 6 performed it in the oxygen pressurized hyperbaric chamber.

Exploring only the English literature, 2 RCTs^12^^,^^13^ and 4 retrospective observational studies^14^, ^15^, ^16^, ^17^ were published (Table 1). All these studies concluded that HBO therapy improved neurological functions compared with the control group and should be considered a support therapy for treating acute SCI. However, several critical issues should be considered: the population, the treatments, including HBO therapy protocol, and follow-up timing. All these factors represent limits for results generalization.

**Table 1:** The table shows the main characteristics of the studies about HBO therapy to treat SCI.

Study (year)	Study type	Number (Control/HBO)	Site of SCI	Surgical time	Drugs	HBO protocol	Score	Conclusions
Sun et al.^12^ (2019)	RCT	38/41	-	-	No mention about timing. Methylprednisolone (30 mg/kg during the first hour, then 5.4 mg/kg/h for 23 h, then 80 mg/day for 7 days), diuretic and neurotrophic agents (ganglioside GM-1, 100 mg/day, and mecobalamin 0.5 mg/day, both for 20 days).	No mention about timing. Pressure: 2.0 ATA; 30 cycles, 115 min/cycle, once a day.	ASIA motor and pain score, Frenkel Grade motor and pain score, before, and on 1st, 3rd, 7th, 10th and 30th treatment day	HBO therapy may reduce the inflammatory reaction that occurs during secondary SCI and promote neurological function recovery.
Feng et al.^13^ (2017)	RCT	20/20	C, T, L	Within 15 days	No mention about timing. Traditional Chinese drugs (Danhong) and neurotrophic agent (Monosialotetrahexosylganglioside sodium, once a day)	No mention about timing. Pressure: 2.0 ATA; 110 min/cycle, once a day, for 8 weeks.	ASIA scores, HAMD, HAMA and FIM, Before and after 8 weeks of treatment.	HBO therapy can significantly relieve depression and anxiety, and improve physical function in the patients with incomplete SCI.
Nekhlopochyn et al.^14^ (2021)	ROS	28/28	C, T, L	From 24 h to 3 days	-	Started on 4th post-operative day. Pressure:1.8 ‒ 2.0 ATA 10 ‒ 12 cycles, 60 ‒ 70 min/cycle	ASIA score on admission and hospital discharge	HBO therapy improves neurological symptoms during the post-operative period, representing a promoting strategy. Further studies are needed to evaluate effectiveness and timing.
Zhang et al.^15^ (2022)	ROS	38/40	C	Within 2 weeks	Started within 8 h from injury, with administration of methylprednisolone (30 mg/kg during the first hour, then 5.4 mg/kg/h for 23 h), diuretic and neurotrophic agent (Mecobalamine, 0.5 mg).	Started as soon as possible. Pressure: 2.0 ATA; 30 cycles, 95 min/cycle, once a day.	ASIA sensory and motor score, Barthel index, before, 1, 3, 6 months and 1, 2, 3 years after surgery.	HBO therapy ameliorated ASIA scores and Barthel indices at 1, 3, 6 months and 1 year. HBO therapy should be started as soon as possible and continued until to 3 months to reach best outcomes.
Tan et al.^16^ (2018)	ROS	11/29	-	4 ‒ 24 h after injury	Started after surgery with administration of methylprednisolone (30 mg/kg during the first hour, then 5.4 mg/kg/h for 23 h), and neurotrophic agents (Monosialo tetrahexosyl ganglioside and mecobalamine, administered whitin 72 hours after injury, 100 mg/die for 3 weeks)	Started on the 1st-3rd post-operative day. Pressure: 2.0 ATA. 30 cycles, 160 min/cycle, once a day.	ASIA scale on day 0, 15, and 30 post-HBO.	HBO therapy ameliorated motor and sensitive functions on 15th and 30th day. HBO showed effectiveness in patients with acute SCI.
Asamoto et al.^17^ (2000)	ROS	21/13	C	-	-	Started within 24 h post-injury. Pressure: 2.0 ATA; 10 cycles, 85 min/cycle, once a day.	Neurological Cervical Spine Scale and ASIA score, before and after HBO	HBO improved outcome in treated patients and should be considered as a support therapy for the treatment of traumatic SCI.

RCT: randomised controlled trial; ROS: retrospective observational study; HBO: hyperbaric oxygen; ATA: absolute atmosphere; ASIA: American Spinal Injury Association; MBI: modified barthel index; HAMD: Hamilton depression scale; HAMA: Hamilton anxiety scale; FIM: functional independence measure; C: cervical; T: thoracic; L: Lumbar.

## 3 Critical issues

### 3.1 Injury site and lesion definition

In the explored literature, injury site was variable or not reported. Furthermore, a clear definition of the lesion, if complete or incomplete, was lacking.

The injury site can represent a limiting factor for the timely start of HBO therapy protocol. After admission, early death rates for SCI range from 4% to 20%^5^^,^^18^, ^19^, ^20^, ^21^, and the level of injury predicts survival rate. Furthermore, patients with severe systemic injuries, traumatic brain injury and suffering from medical comorbidity show an increase in mortality rate.^20^, ^21^, ^22^ Compared with thoracic or lower levels SCI, patients with cervical injuries have a 6.6-fold increased risk of death for C_1_ to C_3_ lesions, a 2.5-fold increased risk for C_4_ to C_5_, and a 1.5-fold increased risk for C_6_ to C_8_.^23^

Patients with incomplete injury without substantial comorbidities or medical complications showed the most significant improvement^20^^,^^24^, with acceptable recovery in the first 6 months^25^. The initial severity and level of injury influence the rates of motor score improvements.^26^, ^27^, ^28^ Among patients with complete SCI (ASIA grade A), only 10% – 15% improve. In patients with an initial ASIA grade B, 54% recover to grade C or D, and 40% regain some ambulatory ability.^29^ Finally, independent ambulation is possible for 62% of patients with an initial ASIA grade C and 97% for those with a grade D.

### 3.2 The treatments

The SCI treatment is articulated in surgery and supporting therapies including drug administration and HBO. The main goals of surgical procedures are the reduction of dislocations, decompression of neural elements and spinal stabilization. Conversely, severe SCI represents a life-threatening condition requiring intensive medical care and continuous monitoring. Consequently, the clinical conditions limit an early surgical approach and the following HBO therapy. Nowadays, there are no evidence-based guidelines regarding the indications for the timing of surgery in SCI.^30^ The management depends on the surgeon's experience and local practice guidelines. Despite these limits, studies demonstrated that patients treated with decompressive surgery within 24 h showed a better outcome than those treated either conservatively or with delayed surgery.^31^

In the context of HBO therapy, in 2 studies^12^^,^^17^, there was no mention of surgical time, and when the time was reported^13^, ^14^, ^15^, ^16^ existed a wide range, from hours to weeks. Furthermore, there is insufficient data about surgical time intervention between control and HBO therapy groups. Weighted analysis, including surgical time intervention, was not performed between the control and treatment groups. Because surgical intervention time influences the outcome, the need to perform as soon as possible HBO therapy could shorten the waiting for surgery. Consequently, the best result found in the HBO group could be the effect of an “early” or “not-to-late” surgical approach.

In all studies, HBO therapy was performed in the hyperbaric chamber at 1.8 – 2.0 absolute atmosphere (ATA) for at least 60 min per cycle. However, wide variability exists about HBO timing and how long the treatment should be continued.

On the first aspect, in 2 studies^12^^,^^13^, there was no mention of HBO therapy timing. In study of Asamoto et al.^17^, HBO treatment started within 24 h post-injury, and Zhang et al.^15^ declared that treatment started “as soon as possible”, but they did not provide further details. Only Nekhlopochyn et al.^14^ and Tan et al.^16^ provided a specific temporal range from the 1st to the 4th postoperative day. However, the first did not provide details about surgery timing, while the second specified that surgery occurred within 4 – 24 h after injury.

The matter is not secondary if we consider HBO as a treatment to reduce secondary injury. The secondary injury begins within minutes and evolves over several hours after the primary injury.^5^^,^^6^ Oedema develops within hours of injury, becomes maximal between the 3rd and 6th day, and begins to recede after the 9th day. Consequently, HBO therapy can be considered a time-sensitive treatment: if possible, it should be started as soon as possible to reach the powerful effects. However, several clinical conditions can limit its timely adoption. Patients with SCI require intensive medical support and continuous monitoring of vital signs, cardiac rhythm, arterial oxygenation, and neurologic signs in the intensive care unit. Furthermore, several systemic complications, especially neurologic, are common in the first days and weeks after trauma. Moving these patients to perform HBO therapy can be challenging, both for the underlying clinical conditions and because the movement can dislocate the bone fragments, thus aggravating spinal cord damage. In this case, the onset of HBO therapy is subject to surgery if the patient's clinical condition allows it.

About the second aspect, how long HBO therapy should be performed, all studies reported a daily session, repeated for a minimum of 10 days up to 8 weeks. Unfortunately, no studies compared different treatment schemes, and data reported in the literature showed that clinical improvement starts during the first 10 days of treatment^12^, continuing to improve up to 6 months after surgery^15^.

Although glucocorticoids and gangliosides were the most common drugs administered in the explored literature^12^^,^^15^^,^^16^, the current evidence suggests against the administration. About glucocorticoids, methylprednisolone is the only treatment that has been suggested in clinical trials to improve neurologic outcomes in patients with acute and nonpenetrating SCI. However, evidence is limited, and its use is debated.^32^ Treatment with glucocorticoids represents an option and not a standard of care because no clinical evidence exists to recommend its use.^33^ Gangliosides are endogenous compounds in cell membranes, with the hypothetical role of protecting nerve cells and promoting axon growth. Two RCTs analysed the effects of GM-1 ganglioside treatment^34^^,^^35^ with discordant results. Based on the available evidence, gangliosides are not recommended to treat SCI.^36^

### 3.3 The follow-up

An important point is the scoring system used for follow-up evaluation and timing. The most used scores in the literature as primary outcomes are ASIA motor and sensory scores and the MBI. Only Feng et al.^13^ evaluated HAMD and HAMA scores because they also analysed the effects of HBO on depression and anxiety in patients with incomplete SCI. In the present review, we focused on ASIA and MBI.

ASIA score measures the injury entity evaluating sensory and motor functions. The assessment of the sensory function includes the ability to sense a sharp object and light touch of both sides 28 dermatomes. The test results were classified into 3 different grades, with 0 indicating the absence of sensory functions or the inability to distinguish between blunt and sharp stimuli, 1 indicating partial dysfunctions, including hypersensitivity, and 2 for normal function. The total maximum score is 112. Regarding motor assessment, each patient's key muscle group is evaluated using the Manual Muscle Test method, with a score ranging from 0 (no contraction or movement) to 5 (active movement against full resistance) points. The higher obtainable score on each side of the body is 50 points (maximum 100 points for one patient).

The ASIA injury classification includes A, B, C, D, and E grades. Grade A refers to complete SCI without sensory and motor function in S4–S5.^37^ Grades B, C, and D belong to incomplete SCI. Grade B retains sensory but not motor function below the injury level, including the sacral region. In Grade C, motor function is preserved below the neurologic level, but the muscle strength of more than half of the key muscles is below Grade 3. Grade D has motor function, and the muscle strength of more than half of key muscles is above Grade 3. Finally, a patient with Grade E has normal sensory and motor functions.

The MBI measures what the patient can do in practice, evaluating the activities of daily living for stroke patients or patients with other disabling conditions. MBI below 15 points usually represents moderate disability, while when below 10 points is a marker of severe disability.

Although these scores are widely used in studies, the different time-points reporting make data analysis and comparisons difficult.

## 4 All we need is protocol

The most important concept is that HBO therapy represents a prevention/treatment strategy for secondary injury. Because the secondary injury begins within minutes and evolves over several hours^5^^,^^6^, with neurologic deterioration over the first 8 – 12 h in patients with an incomplete SCI, HBO therapy should be started as soon as possible according to patients’ clinical conditions and lesion characteristics.

However, a protocol for HBO therapy should consider that (1) not all patients are eligible for early treatment due to their unstable clinical conditions and requirement for continuous monitoring; (2) in the case of an unstable bone lesion, HBO therapy is secondary to surgery because of the risk for further injuries due to patient mobilization.

**Fig. 1:**
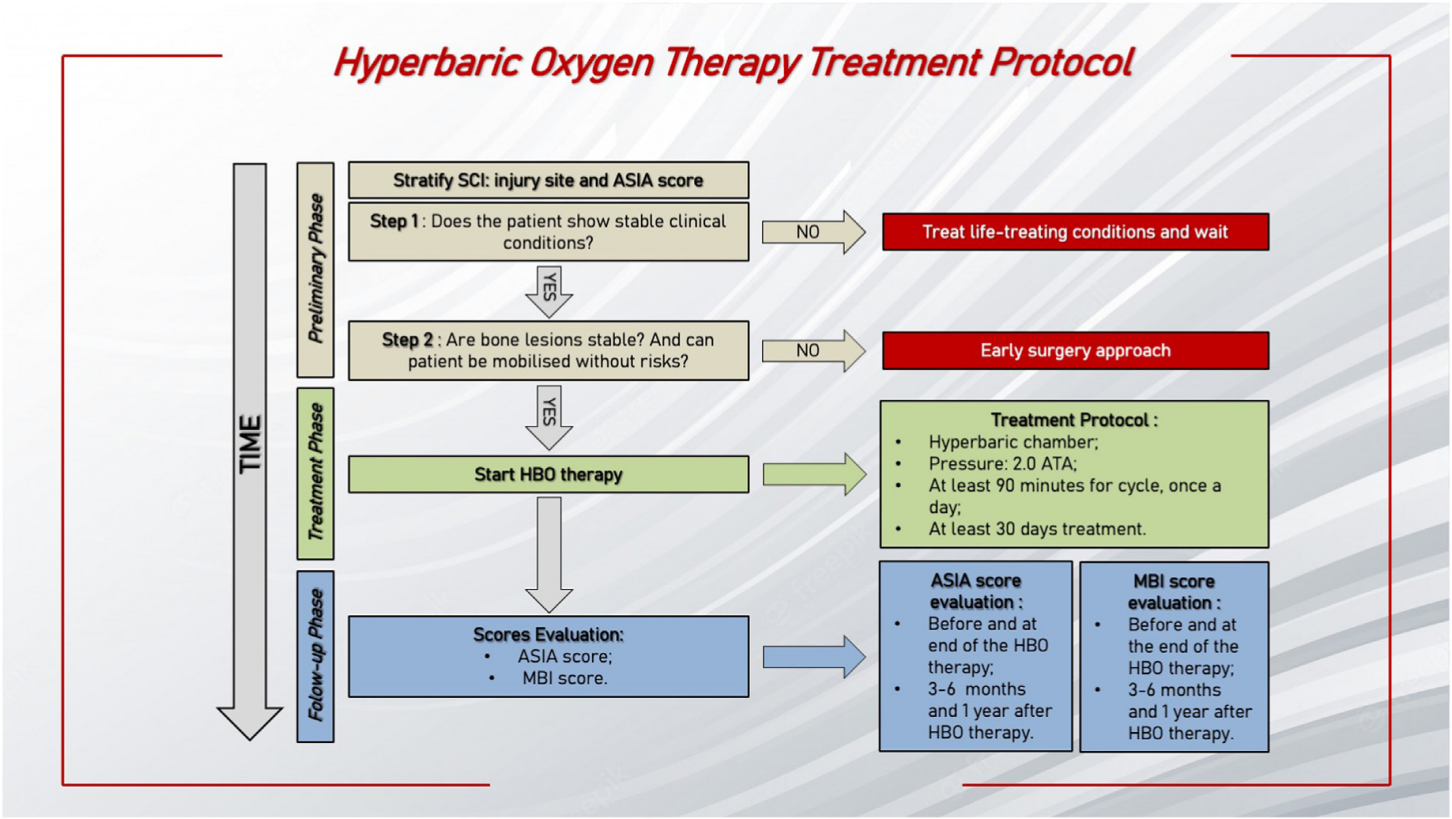
The figure shows the hyperbaric oxygen treatment protocol flowchart. SCI: spinal cord injury; HBO: hyperbaric oxygen; ATA: absolute atmosphere; ASIA: American Spinal Injury Association; MBI: modified barthel index.

Fig. 1 shows the flow chart for the HBO treatment protocol. To add evidence for HBO therapy, this protocol should be adopted for patients with SCI, according to site lesion and ASIA score.

SCI should be classified based on injury site (cervical, thoracic, lumbar) and ASIA score. The preliminary phase consists of 2 steps: (1) evaluation for the presence of life-treating conditions; (2) evaluation for unstable lesions limiting the safe mobilization of the patients. In this phase, HBO therapy can be postponed in case of unstable clinical conditions or the need for surgical treatment.

**Fig. 2:**
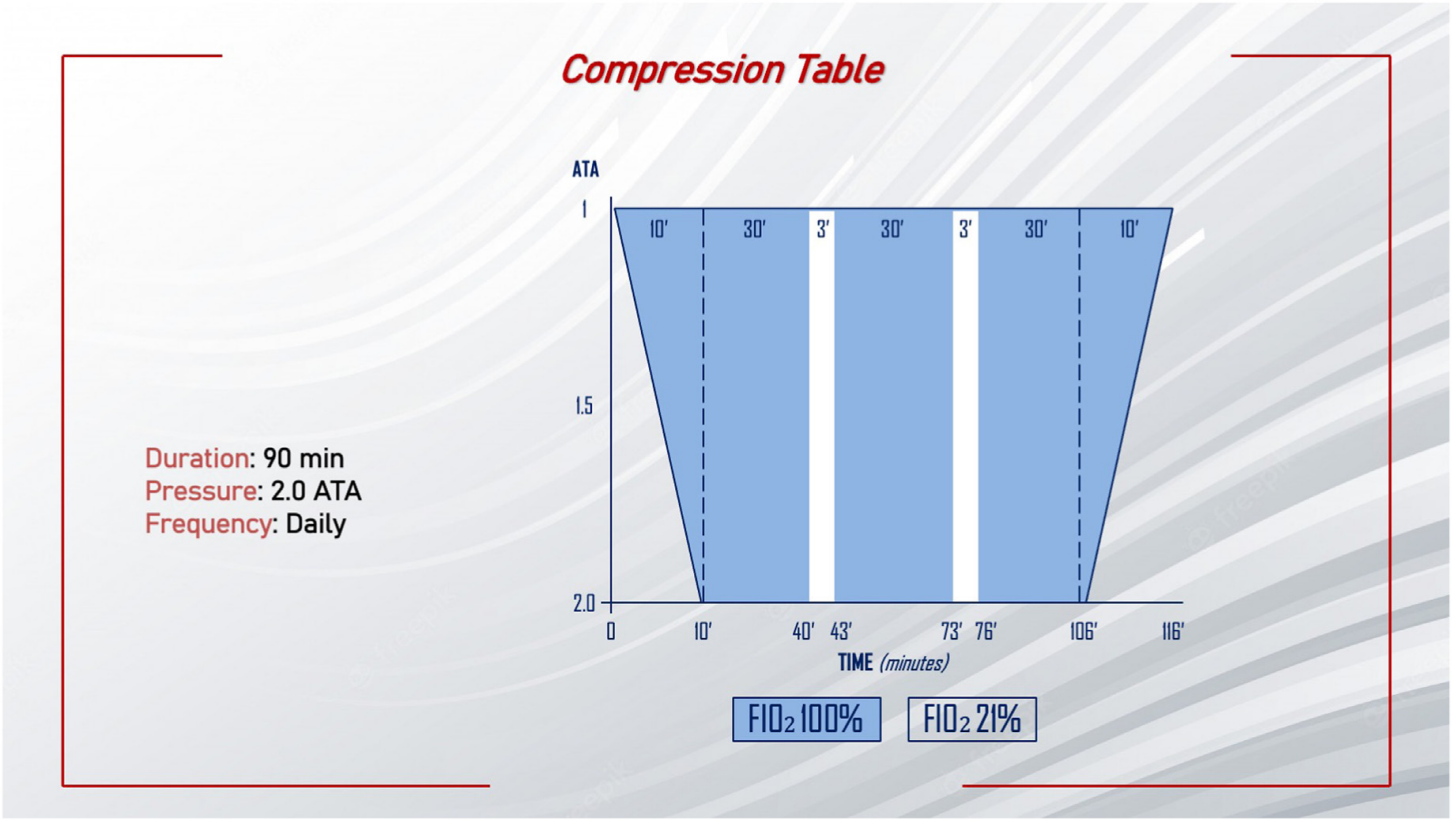
The compression table proposed for the protocol. ATA: absolute atmosphere.

Patients considered eligible can enter the treatment phase. Data reported in the literature suggest that HBO therapy should be performed in the hyperbaric chamber, at 1.8 – 2.0 ATA, for at least 60 min per cycle, once a day, with a duration of at least 10 days. Data showed that the best results are obtained with a pressure of 2.0 ATA.^38^ Exposition to 2.0 ATA for at least 1 h is necessary to saturate the venous blood haemoglobin in humans at rest and another 30 min to saturate the other body tissues. So, a treatment that lasts a shorter period cannot be considered as effectiveness. Fig. 2 shows the compression table for the proposed protocol. The duration of a complete cycle of HBO is 116 min. The first phase (10 min) consists of a pressurization phase until to reach 2 ATA, with a FiO_2_ of 100%. Once the targeted ATA is reached, the treatment starts with 3 cycles lasting 30 min with FiO_2_ at 100%, with a break lasting 3 min with FiO_2_ at 21%. The final decompression phase lasts 10 min with FiO_2_ at 100%.

The next phase consists of the follow-up. Sun et al.^12^ and Zhang et al.^15^ showed that HBO treatment for 30 days improves the ASIA and Frankel scores. In the first 30 days, patients treated with HBO showed a higher improvement in motor function and psychological status than the non-treated group. ASIA score should be used to monitor the patient's clinical improvement after HBO therapy, 3, 6 months and 1 year after treatment. The improvement of quality of life should be monitored using MBI score at 1, 3, 6 months and 1 year after HBO therapy.

Regarding glucocorticoids and gangliosides administrations, the current evidence suggests against their use.

## 5 Conclusion

Despite HBO therapy showing encouraging results in preventing/treating secondary injury related to SCI, the heterogeneity of studies limits the generalization of the results. There is a need for a rationale and shared protocol to standardize as much as possible the population to be studied, the treatment to be adopted, and the outcomes to be evaluated.

In the future, well-designed RCTs are needed to clarify the role of HBO therapy as a strategic tool to prevent/reduce secondary injury in SCI and evaluate its effectiveness based on a standardized treatment protocol. However, we hope that adopting the proposed protocol can reduce the risk of bias and drive future studies.

## Funding

Nil.

## Ethical statement

Not applicable.

## Declaration of competing interest

None.

## Author contributions

All authors were involved in drafting the manuscript and reviewing and approving the published version.
